# Gastric Adenocarcinoma of the Fundic Gland Type Treated by Endoscopic Mucosal Resection: A Case Report and Review of the Literature

**DOI:** 10.1155/2016/8646927

**Published:** 2016-11-22

**Authors:** Eleanor Lewin, Philip Daroca, Sanjay Sikka, Tong Wu, Yukihiro Nakanishi

**Affiliations:** ^1^Department of Pathology, Tulane University School of Medicine, New Orleans, LA, USA; ^2^Department of Medicine, Tulane University School of Medicine, New Orleans, LA, USA

## Abstract

Gastric adenocarcinoma of the fundic gland type (GA-FG) is a rare entity that has only recently been described and defined. There is ongoing controversy regarding the malignant potential of this lesion. We report the case of a GA-FG in a 49-year-old Caucasian man who was referred to endoscopy for management of an incidentally found gastric polyp. Endoscopy showed a single polypoid lesion in the gastric fundus which was successfully removed with endoscopic resection. Grossly, the polyp measured 1.1 cm in greatest dimension. Microscopic examination showed irregularly branched neoplastic glands covered with a nonneoplastic foveolar epithelium. The continuity between the neoplastic glands and the fundic glands is clearly identified, indicating the tumor arose from the fundic glands. The tumor cells exhibited occasional oxyntic cytoplasm with enlarged atypical nuclei. The tumor invaded the submucosa with complete disruption of the muscularis mucosae and mild lymphocytic and fibroblastic stromal reaction. No necrosis, mitosis, or lymph-vascular invasion was identified. Although some authors have proposed reclassification of GA-FGs as oxyntic gland polyps/adenomas, in light of several reported cases with submucosal invasion as well as lymphatic invasion, we maintain that this neoplasm is best categorized as an extremely well-differentiated adenocarcinoma to reflect its invasive potential.

## 1. Introduction

Gastric adenocarcinoma (GA) has traditionally been divided into two main categories: intestinal-type and diffuse-type [1]. The intestinal-type is presumed to stem from chronic inflammation, most often from infection by* Helicobacter pylori*, resulting in intestinal metaplasia with eventual progression to dysplasia and finally to carcinoma; thus, intestinal-type GA is presumed to have begun as a well-differentiated adenocarcinoma with an intestinal immunophenotype. Diffuse-type GA, on the other hand, is poorly differentiated at the onset, typically showing tumor cells filing through a fibrotic and inflamed stroma without gland formation; this subtype of GA is believed to arise directly from gastric epithelium without intervening metaplasia and therefore demonstrates a gastric immunophenotype.

In recent years, however, cases of well-differentiated gastric adenocarcinoma with fundic gland morphology and immunophenotype have been described and termed “well-differentiated gastric adenocarcinoma of the chief cell or fundic gland type” (GA-FG) [2–11]. These lesions demonstrate a variety of endoscopic characteristics but, histologically, all show tightly packed to dilated glands composed of oxyntic tumor cells. Nuclear pleomorphism is mild and mitotic activity is not evident. Due to this seemingly innocuous profile, there has been suggestion to reclassify GA-FGs as oxyntic gland polyps or adenomas [11]. In this paper, we report a rare case of GA-FG invading the submucosa with complete disruption of the muscularis mucosae and mild lymphocytic and fibroblastic stromal reaction, confirming the diagnosis of GA-FG. Most of GA-FGs have been described in Japanese patients [2–10]. This is the second report of GA-FG from the United States [11].

## 2. Case Report

**Figure 1:**
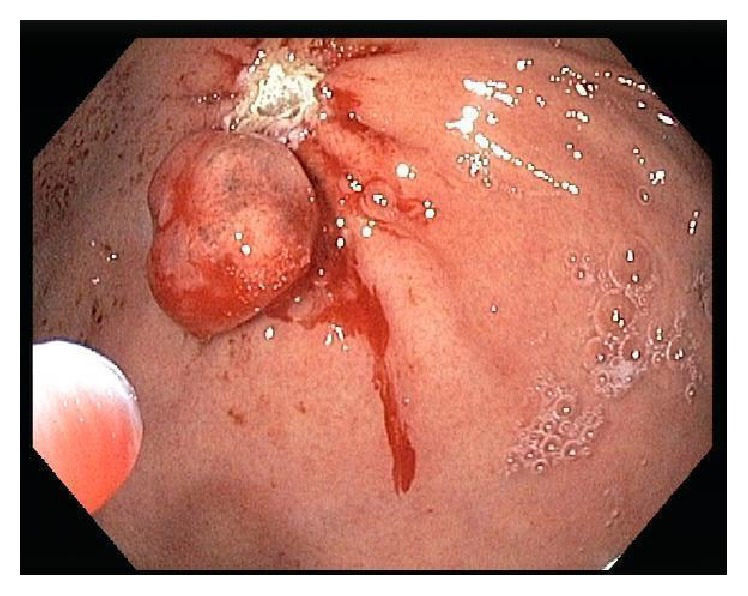
The polypoid lesion was identified as a whitish elevation covered with smooth surface. The polyp was successfully removed with endoscopic resection.

**Figure 2:**
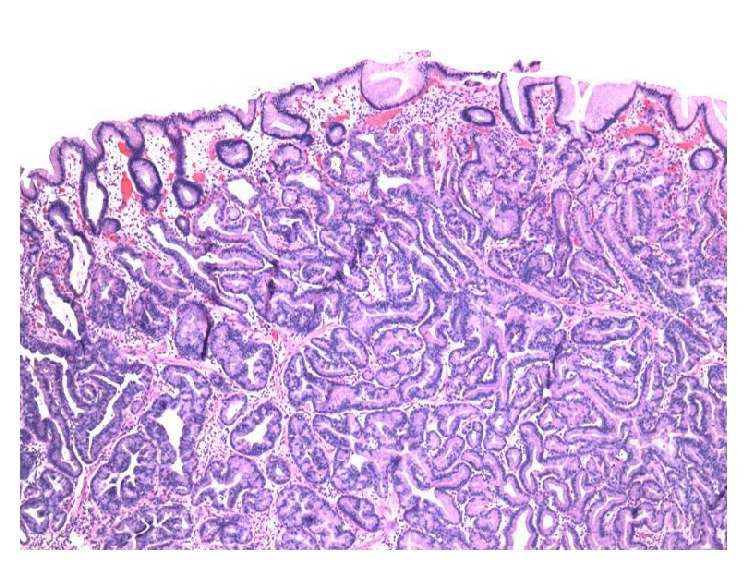
Irregularly branched neoplastic glands covered with the nonneoplastic foveolar epithelium (H&E ×40).

**Figure 3:**
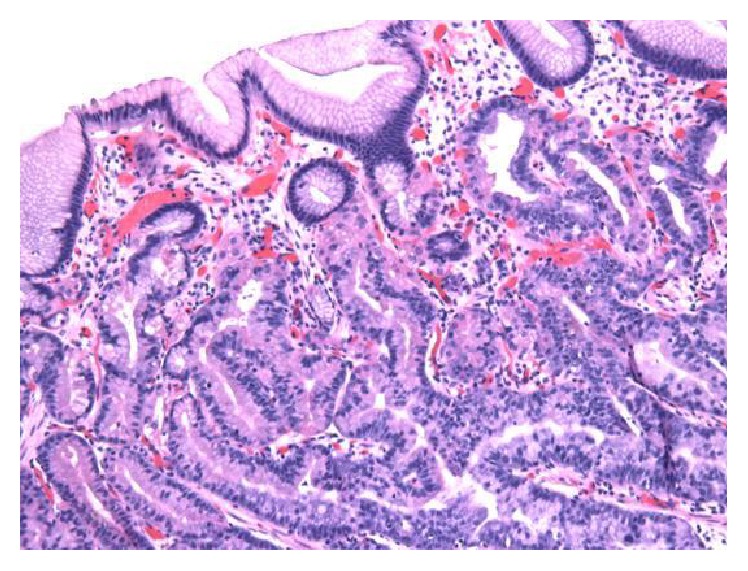
Continuity between the neoplastic glands and the fundic glands, indicating that the tumor arouse from the fundic gland (H&E ×100).

**Figure 4:**
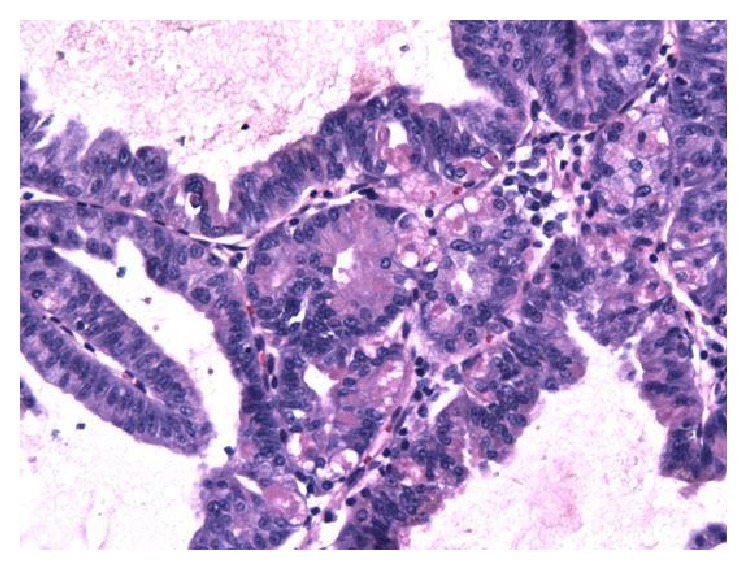
Occasional neoplastic cells showing oxyntic cytoplasm (H&E ×200).

**Figure 5:**
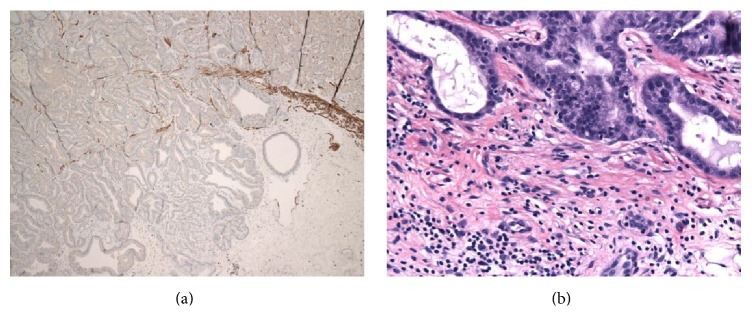
Submucosal invasion with complete disruption of the muscularis mucosae (a) (desmin immunostain ×40) and mild lymphocytic and fibroblastic stromal reaction (b) (H&E ×200).

**Figure 6:**
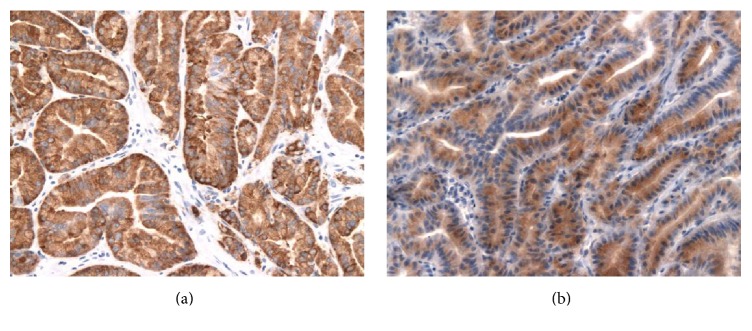
The neoplastic glands are diffusely reactive for MUC6 (a) (MUC6 immunostain ×200) and pepsinogen-I (b) (pepsinogen-I immunostain ×200).

The patient was a previously healthy 49-year-old Caucasian man who was referred to endoscopy for management of an incidentally found gastric polyp. Endoscopy showed a single polypoid lesion measuring 1.0 cm in the gastric fundus. The polypoid lesion was identified as a whitish elevation covered with smooth surface. The endoscopic features of the polypoid lesion were similar to those of submucosal tumor. No biopsy was taken prior to endoscopic resection. No information about the previous biopsy result at outside institution was available. The polyp was successfully removed with endoscopic resection (Figure 1). Grossly, the polyp measured 1.1 cm in greatest dimension. Microscopically, the polyp was composed of irregularly branched neoplastic glands covered with the nonneoplastic foveolar epithelium (Figure 2). Continuity between the neoplastic glands and the fundic glands was clearly identified, indicating that the tumor arose from the fundic glands (Figure 3). The tumor cells exhibited mildly enlarged atypical nuclei with occasional cells showing oxyntic cytoplasm (Figure 4). The tumor invaded the submucosa with complete disruption of the muscularis mucosae and demonstrated mild lymphocytic and fibroblastic stromal reaction (Figures 5(a) and 5(b)). No necrosis, mitoses, or lymph-vascular invasion was identified. No chronic gastritis or intestinal metaplasia was seen in the background mucosa. Resection margins were negative for neoplasm. Immunohistochemistry demonstrated that the neoplastic glands were strongly and diffusely reactive for MUC6 and pepsinogen-I (Figures 6(a) and 6(b)) and nonreactive for MUC2 and MUC5AC, confirming a gastric immunophenotype. Ki-67 immunostaining demonstrated unevenly distributed variable expression. The Ki-67 labeling index in the area with the highest positivity was approximately 20%. No overexpression of p53 protein or nuclear positivity of beta-catenin was identified. The immunohistochemistry for* Helicobacter pylori* was negative. All immunostains have working controls. These results confirmed the diagnosis of GA-FG.

## 3. Discussion

Gastric adenocarcinomas of the fundic gland type (GA-FGs) have been characterized as neoplastic lesions arising directly from gastric mucosa without intervening intestinal metaplasia [2–11]. Endoscopically, GA-FGs may appear as submucosal nodules or as flat or depressed lesions; they are usually pale whitish and often show dilated surface vasculature [8–10]. Microscopically, the tumor cells mimic benign fundic gland epithelium and show abundant oxyntic cytoplasm with only mild nuclear atypia [2–11]. These lesions often show disruption of the muscularis mucosa with submucosal invasion but only rarely demonstrate lymph-vascular invasion [6, 9]. GA-FGs maintain a gastric immunophenotype, with features overlapping between fundic gland mucous neck cells and chief cells [2–11]. Our case well demonstrates the above-described histologic and immunohistochemical features of GA-FGs. The neoplastic glands underlie an unremarkable foveolar epithelium and show clear penetration through the muscularis mucosa into the submucosal layers. The neoplastic glands are reactive for MUC6 and pepsinogen-I and nonreactive for MUC2 and MUC5AC, consistent with gastric rather than intestinal differentiation. As with most of other described cases [5, 10], there was no evidence of* H. pylori* infection, either on routine histology or by immunohistochemistry.

Due to above-mentioned seemingly innocuous profile including mild nuclear pleomorphism and lack of mitotic activity, there has been suggestion to reclassify GA-FGs as oxyntic gland polyps or adenomas [11]; however, our case clearly showed invasion into the submucosa with complete disruption of the muscularis mucosae and demonstrated mild lymphocytic and fibroblastic stromal reaction. Previous publications have also reported submucosal invasion as well as lymphovascular invasion [6, 9]. Given these features, we agree with earlier authors in their description of this entity as a carcinoma, albeit one with low metastatic potential [2–10].

Given the unique growth pattern of dilated neoplastic glands covered with the nonneoplastic foveolar epithelium as well as mild nuclear pleomorphism and lack of mitotic activity, the differential diagnosis includes fundic gland polyp, dysplasia in fundic gland polyp, gastritis cystica profunda, well-differentiated neuroendocrine tumor (carcinoid tumor), and reactive epithelial changes. The presence of mild nuclear pleomorphism, irregular branching of neoplastic glands, and submucosal invasion as well as immunohistochemical staining could help establish the diagnosis.

Most GA-FGs are less than 1.5 cm and are amenable to endoscopic resection by either mucosal resection or submucosal dissection [2, 5, 9, 10]. In our patient, the lesion was fully excised by endoscopic mucosal resection. Margins were negative and no recurrence has been reported more than one year later. In one case series including ten cases from the United States, only one case had persistence of tumor following endoscopic resection [11], and this was due to incomplete initial removal of tumor; all other patients with available follow-up data showed no evidence of disease at the most recent follow-up visit after 6 to 57 months from the removal of tumor [2, 5, 9–11]. One case report, however, has discussed more aggressive lesion with a large tumor measuring 4.4 cm in greatest dimension and extensive lymph-vascular invasion [6].

Most published cases of GA-FGs have come from Japan [2–10]. The reason for this is not clear; it may be speculated that Japan has the high incidence of stomach cancer [12] and that gastric cancer screening has been conducted nationwide for all residents aged 40 years and over [13]. Our case is the second report of GA-FG from the United States [11]. Although most of GA-FGs are described in Japanese patients, GA-FGs can arise in various ethnic groups including Caucasian, Hispanic, African American, Chinese, and Korean [11, 14].

The pathogenesis of GA-FG is uncertain and may be different from that of conventional gastric cancer. One previous study reported that GA-FGs were characterized by nuclear beta-catenin accumulation and mutation of CTNNB1 or AXIN gene, suggesting activation of the Wnt/beta-catenin pathway [15]. More recent studies suggested that GNAS mutation might occur in a small subset of gastric adenocarcinomas of the fundic gland type as an alternative mechanism of activating the Wnt/beta-catenin signaling pathway [4]. In our case, however, no nuclear beta-catenin accumulation was identified. Further investigation is needed to elucidate the pathogenesis of this unique variant of nonaggressive gastric adenocarcinoma.
